# Anticoagulation Management in V-V ECMO Patients: A Multidisciplinary Pragmatic Protocol

**DOI:** 10.3390/jcm13030719

**Published:** 2024-01-26

**Authors:** Ana Bento Rodrigues, Anabela Rodrigues, Catarina Jacinto Correia, Gustavo Nobre Jesus, João Miguel Ribeiro

**Affiliations:** 1 Serviço de Medicina Intensiva, Centro Hospitalar Universitário Lisboa Norte, 1649-028 Lisboa, Portugal; gustavonjesus@gmail.com (G.N.J.); jmribeiro@chln.min-saude.pt (J.M.R.); 2 Clínica Universitária de Medicina Intensiva, Faculdade de Medicina de Lisboa, 1649-028 Lisboa, Portugal; 3 ECMO Referral Centre, Intensive Care Department, Centro Hospitalar Universitário Lisboa Norte, 1649-028 Lisboa, Portugal; 4 Serviço de Imuno-Hemoterapia, Blood Transfusion Department, Centro Hospitalar Universitário Lisboa Norte, 1649-028 Lisboa, Portugal; anabela.rodrigues@chln.min-saude.pt (A.R.); cfjc@campus.ul.pt (C.J.C.)

**Keywords:** ECMO, anticoagulation, protocol, bleeding, thrombosis, testing

## Abstract

(1) Background: Extracorporeal membrane oxygenation (ECMO) is a complex procedure affecting both the risk of thrombosis and bleeding. High-quality data to personalize anticoagulation management in ECMO are lacking, resulting in a high variability in practice among centers. For this reason, we review coagulation methods and monitoring and share a pragmatic proposal of coagulation management, as performed in our high-volume ECMO Referral Centre; (2) Methods: We revised the anticoagulation options and monitoring methods available for coagulation management in ECMO through PubMed search based on words including “anticoagulation,” “coagulation assays,” “ECMO,” “ELSO,” and “ISTH”; (3) Results: Actual revision of the literature was described as our routine practice regarding ECMO anticoagulation and monitoring; (4) Conclusions: No coagulation test is exclusively predictive of bleeding or thrombotic risk in patients undergoing ECMO support. An approach that allows for a tailored regimen of anticoagulation (regardless of agent used) and monitoring is mandatory. To accomplish this, we propose that the titration of anticoagulation therapies should include multiple laboratory tests, including anti-Xa, aPTT, ACT, viscoelastic tests, AT levels, platelet count, fibrinogen, and FXIII levels. Anticoagulation regimens should be tailored to a specific patient and personalized based on this complex array of essays.

## 1. Introduction

The implementation of veno-venous extracorporeal membrane oxygenation (V-V ECMO) for the treatment of respiratory failure has been established after the H1N1 Influenza and SARS-CoV-2 pandemics [1,2,3,4]. Albeit the advent of miniaturized extracorporeal circuits, ECMO remains a complex procedure associated with potential risks and life-threatening adverse events. Recognizing the risk of bleeding and thrombosis is paramount to defining strategies to reduce its occurrence and provide safety for the patient under ECMO support [1]. Protocol-driven coagulation management should be implemented in every ECMO center, balancing the risk of venous or extracorporeal circuit thrombosis and the risk of bleeding. Given the high incidence of thrombotic events, it seems yet unfeasible to overcome the need for anticoagulation recommendation [1,2]. Nevertheless, it should be recognized that some patients pose a high risk for hemorrhagic events, and some have an absolute contraindication to antithrombotic therapy (for instance, severe trauma, alveolar hemorrhage, or severe thrombocytopenia).

Additionally, it should be recognized that the provision of ECMO significantly impacts the balance of hemostasis. Clot formation emerges due to a complex and multifactorial process driven by the extracorporeal biomaterial-mediated activation of coagulation, complement, and inflammatory systems; increased platelet activation and release of prothrombotic granules and microparticles further unbalance hemostasis [3,4]. Concomitantly, the ECMO circuit can induce bleeding associated with thrombocytopenia, platelet dysfunction, shear-mediated loss of high molecular weight von Willebrand factor (VWF) multimers, hypofibrinogenemia, coagulation factor (F) consumption (including FXIII) and inflammation [4,5]. Hemolysis is expected by usage of an extracorporeal device [6]. This form of “rebalanced hemostasis” is exceedingly difficult to predict and has thus driven efforts to refine both ECMO technology and clinical practice [4]. Clot formation and fibrin deposition on the oxygenator are the main causes of lower technique efficiency and durability of the filter and can potentiate mortality [7]. The lack of high-quality data to guide anticoagulation management in ECMO patients results in high variability in practice among centers [8,9,10]. For this reason, we review and share a proposal for coagulation management, as performed in our ECMO-referral Centre.

## 2. Materials and Methods

We revised the anticoagulation options and monitoring methods available for coagulation management in ECMO through a PubMed search based on words including “anticoagulation,” “coagulation assays,” “ECMO,” “ELSO,” and “ISTH.” From sixty manuscripts, we have chosen the most recent 35 to include in this review, translating the most recent research on the field in the last 10 years.

## 3. Relevant Sections

### 3.1. Choosing Anticoagulation

#### 3.1.1. Unfractionated Heparin (UFH)

UFH is still the chosen anticoagulant recommended in recent guidelines [8,10,11,12]. Thrombotic and hemorrhagic patient risks and ECMO modality should be considered, with individualized targets.

UFH binds to antithrombin (AT), causing activated factor X (FXa) and thrombin (FIIa) inhibition. It has a half-life of 60 to 90 min in adults, explaining the need to interrupt timely for potentially invasive procedures [11].

UFH has several advantages, such as low cost, easy accessibility, and being quickly reversible with protamine sulfate [11] (1 mg neutralizes 100 units of UFH). Concerns regarding its use are binding to plasma proteins, endothelial cells, and macrophages interfering with its action and monitoring [11]. There is also the risk of heparin-induced thrombocytopenia (HIT) and heparin resistance (HR) [8,11] (Section 3.3—Anticoagulation complications). Regular assessment of AT level and supplementation is suggested in ECMO patients due to its consumption [12].

#### 3.1.2. Bivalirudin (Direct Thrombin Inhibitor—DTI)

Direct thrombin inhibitors, such as bivalirudin and argatroban, reversibly bind to thrombin [11] and are alternatives to UFH, mainly when complications such as HIT or HR occur [8,9,11,12]. Bivalirudin, the only DTI available in our Centre, has a more predictable pharmacokinetic profile, with a half-life of 25 min in adults, requiring a shorter interruption time before procedures [9,11]. DTI has the advantage of not requiring antithrombin therapeutic levels [11] and having a fast washout period. As a disadvantage of DTI, unlike UFH, there is no specific antidote for reversal, and there is the need to adjust dosage in patients with hepatic and renal failure due to the risk of supratherapeutic dosage [11]. In patients with blood stasis, a lack of efficacy with clot formation might ensue [11]. In addition, the limited availability of specific laboratory monitoring and higher cost are considered disadvantages [11].

### 3.2. Monitoring and Available Laboratory Testing

#### 3.2.1. Activated Partial Thromboplastin Time (aPTT)

- Recommended goal for monitoring: >1.5 to 2 times control (no randomized trial for ECMO patients) [11,12,13].

aPTT is a coagulometric plasmatic test by turbidimetry, measuring time from factor FXII activation until fibrin formation after calcium (Ca^2+^) addition and phospholipidic exposure with contact with coagulation activator [11,13]. It is widely available and routinely performed throughout most centers [11,12,13]. It is the standard method for assessment of anticoagulation with UFH [8,11,13] and can also be used to monitor DTI usage [8,9,12,13]. aPTT at higher doses of DTI shows a nonlinear dose response [11].

The principle that supports this method assumes a linear correlation of the patient’s baseline aPTT to the reference range [11]. In critical patients, the baseline aPTT is often altered (i.e., due to inflammatory status or coagulation factor deficiency), limiting UFH monitoring [11] or requiring additional tests for accurate anticoagulation testing.

As aPTT evaluates contact activation in the intrinsic pathway, there is interference of acute phase inflammation markers such as FVIII and fibrinogen [8,10,11], often elevated in the critical patient. This masks the anticoagulation effect and shortens aPTT [11]. There will be misinterpretation of the need to increase anticoagulation. On the other hand, in these patients, interference with inhibitors, such as lupus anticoagulant (LA), will increase aPTT, overestimating anticoagulation. Interference is dependent on the reagents used in the laboratory for aPTT monitoring, with non-sensible LA reagents available [13]. Limitations with this approach are related to local-specific aPTT reagents/lot and coagulometer. aPTT can also be influenced by drugs, hematocrit, acute phase reactants, abnormalities in clotting factors (FXII level), AT level, high C-reactive protein, hyperbilirubinemia, and hyperlipidemia [1,9]. All these issues must be considered before implementing target aPTT values in clinical practice.

Finally, there is intra and inter-patient variability that can increase with repeated aPTT monitoring, blood drawings, and dosage adjustments [11]. Consequently, the anti-Xa assay for UFH monitoring is used in many situations [11].

Correlation between aPTT and anti-Xa activity for heparin monitoring has been demonstrated, with some discordant measurements [8,9,13]. For this reason, we suggest not using aPTT alone in assessing the adequacy of anticoagulation with UFH, namely in cases of heparin resistance [8].

#### 3.2.2. Anti-Xa Activity (Anti-Xa)

- Anti-Xa activity goal for anticoagulation with UFH monitoring in the ECMO patient: 0.3–0.7 U/mL [13,14].

Anti-Xa activity is a plasmatic chromogenic test using absorbance to measure the heparin effect [8], evaluating AT catalysis and inhibition of FXa activity [11,13]. It is only used for monitoring FXa inhibition [8,13] since it solely measures the chemical reaction between the UFH-AT complexes [11] and is increasingly used to measure the heparin effect [8,11]. However, anti-Xa levels do not evaluate clot formation [9].

Unlike the activated coagulation time (ACT) and aPTT methods, the anti-Xa assay is unaffected by coagulopathy, thrombocytopenia, or dilution and best represents the overall heparin anticoagulation level [13].

However, if plasma is opaque or pigmented (hyperbilirubinemia > 6.6 mg/dL, hypertriglyceridemia > 360 mg/dL, hemolysis with high free hemoglobin level), underestimation of UFH anticoagulation is possible [8,10,12]. Anti-Xa can also be influenced by AT deficiency [9]. In addition, calibration for each heparin type is needed [11].

Anti-Xa assay seems to have a better correlation with heparin levels when compared with aPTT or ACT in pediatric ECMO and adult extracorporeal life support (ECLS) patients [8,9,12]. However, when compared with ACT and aPTT, anti-Xa assays are more expensive and not accessible in all hospitals [13]. We suggest daily anti-Xa monitoring as a mainstay of a multimodal coagulation management protocol in patients under UFH.

#### 3.2.3. Activated Coagulation Time (ACT)

- ACT goal for anticoagulation monitoring in ECMO patients is 180–200 s [8,9,13].

ACT is a bedside point-of-care test that uses whole blood as a sample [9,13]. It measures the time until fibrin clot formation after adding clotting activators [11,13]. ACT provides a physical examination of the blood but does not measure clot strength [11]. It permits very fast results, contributing to real-time anticoagulation adjustments [13]. ACT evaluates contact activation/intrinsic coagulation inhibitors by heparin or DTI [9].

Several factors can affect ACT results, such as thrombocytopenia, platelet dysfunction and inhibitors, hypofibrinogenemia, coagulation factor deficiency, hypothermia, hemodilution, anemia, and technical factors [8,10,11,13]. Different ACT machines yield different results, which is why they cannot be used interchangeably [11]. Each center must preferably use the same machine to warrant validated results.

ACT is not licensed to monitor DTI, although it can be used to monitor the anticoagulation effect and trends after reaching the anticoagulation target [11]. In UFH usage, ACT has been approved for monitoring since starting anticoagulant therapy and is widely used in cardiac and vascular surgeries.

However, the correlation between the anticoagulation target and UFH dosage in ECMO patients is still questionable in the literature, as is the correlation with other control methods [8,9,13]. ACT has a poor correlation with heparin concentrations within the dose range typically used for ECMO [8,13]. ACT is an unreliable tool for monitoring UFH during ECLS in adults [8]. The correlation of ACT with anti-Xa and aPTT is frequently discordant [9]. Nevertheless, it is worth noting that most hospitals use ACT methods for routine coagulation monitoring in ECMO [13]. We suggest using either aPTT or ACT daily to monitor UFH anticoagulation.

#### 3.2.4. Viscoelastic Testing (VET)

VETs are point-of-care tests on whole blood samples used when fast and goal-directed therapy is needed. These are qualitative tests that can monitor global hemostasis, giving information regarding clot formation, stability, and lysis; hypercoagulability (eventually predicting thrombotic risk); hypo or hyperfibrinolysis [15].

VET shows a global perspective of hemostasis, measuring the start of clot formation (CT—clotting time), firmness of clot (clot strength, amplitude—considering the contribution of fibrinogen and platelets), and clot stability (evaluation of fibrinolysis, which is extremely relevant in patients under ECMO and for antifibrinolytic therapy monitoring) [16].

It is also possible to measure the anticoagulation effect with UFH in a semi-quantitative manner using two simultaneous specific tests (e.g., INTEM and HEPTEM in rotational thromboelastometry). There are randomized controlled trials regarding VET usage in ECMO patients that show a good predictive value for hemorrhage and thrombosis, being a valuable test for UFH anticoagulation monitoring in ECMO or left ventricular assist device (LVAD) [13,15].

VETs are often used for bleeding management evaluation rather than anticoagulation monitoring [9]. Although these tests appear to provide a real picture of the complex anticoagulation and coagulation system, important disturbances of primary hemostasis, such as the acquired von Willebrand deficiency, would not be reflected in these assays [17].

VET devices require calibration at regular intervals and skilled operators [15]. The sensitivity of the reagents differs between different manufacturers and sets of reagents [15]. We suggest a VET analysis as part of a multimodal coagulation management protocol.

#### 3.2.5. Dilute Thrombin Time (dTT)

dTT is a coagulometric plasmatic test based on thrombin time (TT). dTT and other tests, such as the Ecarin Chromogenic assay (ECA) and Ecarin Clotting Time (ECT), can be used to measure DTI in a wide range of concentrations. However, these are not widely available [11,12]. Compared with aPTT, dTT has increased sensitivity to monitor DTI usage [11,12], being useful in ECMO patients under bivalirudin anticoagulation [12]. If the baseline aPTT levels are abnormal, a dTT can be used [12]. A nomogram to correlate dTT with serum bivalirudin is available [18]. Its main limitation is its scarce availability and cost [12].

#### 3.2.6. Antithrombin (AT)

- AT goal for adults: 80–120% [8,10].

Antithrombin (AT) plays a central role in the anticoagulation system through irreversible inhibition of multiple clotting factors (thrombin, FXa, IXa, XIa, XII, tissue plasminogen activator, plasmin, and kallikrein) [8]. AT also has important anti-inflammatory attributes [8]. In ECMO patients, underlying illness and blood circuit interaction alter the hemostatic balance with AT consumption, which is the reason why timely assessment of AT activity should be considered [10,14].

However, more evidence is needed before recommending routine monitoring and supplementation [10,11]. The International Society of Thrombosis and Hemostasis (ISTH) suggests AT monitoring in patients with thrombosis [12]. Although current data do not support routine AT repletion [12], we recommend in patients with thrombotic events replacement of AT if the level is under 80%. In addition, in the case of anticoagulation with heparin, regular measurement and supplementation, if AT is under 80%, are suggested.

### 3.3. Anticoagulation Complications

#### 3.3.1. Heparin Resistance (HR)

HR is present when an unusually increasing dosage of heparin is needed to reach the therapeutic goal [8,10], identified by anticoagulation monitoring (ACT, aPTT, anti-Xa) or thrombosis occurrence [9,14]. HR is defined as >35,000 units/day of UFH [9,14] or, more recently, as a UFH perfusion rate of >25 U/Kg/h to achieve anticoagulation [9] or >500 U/Kg in cases of cardiopulmonary bypass [14]. However, it does not consider factors possibly influencing the heparin efficacy, e.g., the body mass index, sex, prothrombotic states (COVID-19, sepsis), or AT deficiency [14]. The fluctuation of heparin response among patients has a pharmacokinetic and biochemical basis [14]. Checking an anti-Xa level can be helpful when high doses of heparin do not achieve the desired aPTT or ACT values [9].

Heparin binds to antithrombin (AT) to form the heparin-AT complex, causing inhibition of FXa activity and, to a lesser extent, of FIIa activity [8,13].

Using these principles, we understand and define the causes of heparin resistance (Table 1):

- Pseudo-heparin resistance: occurs when high FVIII and/or fibrinogen levels interfere with aPTT measurement, giving falsely low results and leading to misinterpretation of low heparin efficiency.
- Antithrombin (AT) deficiency: is a common cause of HR [14]. Heparin works by binding to AT so that low levels will cause an effective low heparin effect. Acquired AT deficiency can be secondary to liver disease, sepsis, nephrotic syndrome, malnutrition, acute thrombosis, increased consumption during bleeding or disseminated intravascular coagulation, associated with extracorporeal circuits such as ECMO, and the use of heparin [10,14]. ECMO-related AT deficiency is frequently seen upon ECMO initiation and may be attributed to reduced synthesis plus accelerated consumption [14].
- Low heparin concentration: especially due to the binding of acute phase proteins that change the pharmacokinetics and volume of distribution of the drug. Systemic inflammation increases the production of proteins that bind to heparin—e.g., PF4, causing lower heparin concentration in blood with lower anticoagulant effect and frequent dosage adjustments.
- Multifactorial: Combination of the causes mentioned above.

HR is common in intensive care settings, mainly in severe disease states associated with high systemic inflammatory expression. Management of HR includes increasing the heparin administration dose to bind all available AT [8] and to achieve the goal of anti-Xa level (0.3–0.7 UI/mL) [14], AT supplementation [8,14] and/or using a monitoring method that is not affected by acute phase reactant proteins [14]. In the case of persisting HR, anticoagulation with DTI can be successfully employed [14].

#### 3.3.2. Heparin Induced Thrombocytopenia (HIT)

HIT is an immune, non-bleeding complication caused by antibodies that bind to the complex formed by heparin and platelet factor 4 (PF4). It is frequent (8 to 50%), but clinical manifestations due to thrombocytopenia or thrombosis are rare (0.2 to 3% in heparin exposure) [20].

There is a temporal association of thrombocytopenia with the time of starting anticoagulation, with platelet levels < 150 × 10^9^/L or a sudden drop of platelet count around 30 to 50% of basal levels. Moderate thrombocytopenia (50–70 × 10^9^/L) is usually seen with no bleeding diathesis. Severe thrombocytopenia (<20 × 10^9^/L) can occur as a manifestation of a fulminant thrombotic event or consumption coagulopathy [21,22].

Even in isolated thrombocytopenia, the risk of thrombosis is around 20 to 50% [23]. Thrombotic events such as skin necrosis can be present in HIT, even in the absence of thrombocytopenia, and thrombotic manifestations can occur in any vascular territory, more frequently in vascular access sites [21,24].

**Figure 1:**
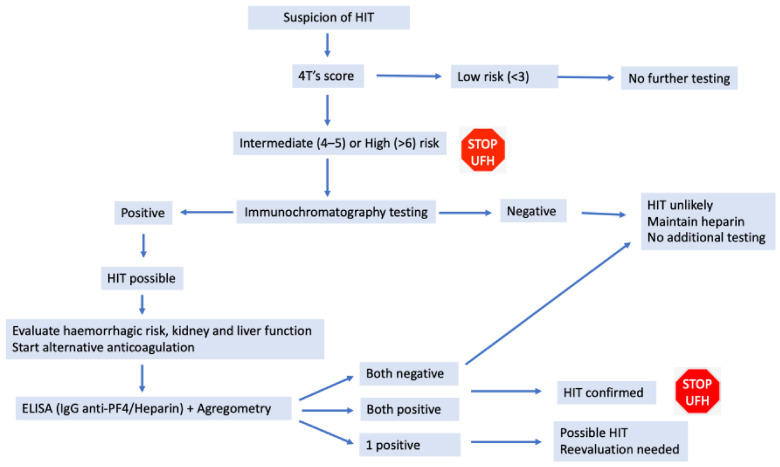
Algorithm of diagnosis of Heparin-induced thrombocytopenia [10,27]. HIT: Heparin-induced thrombocytopenia; Ig: Immunoglobulin; PF4: platelet factor 4.

Diagnosis: The 4Ts score (Table 2) assesses the risk of HIT and defines it as low, intermediate, or high [25]. Laboratory testing (Figure 1) with immunochromatography assay (Stago STiC^®^) detects the presence of antibodies. This is a presumptive test since it has a high prevalence of false positive results [26]; if the test is negative, no further testing is needed. In case of positive results, a confirmatory test such as ELISA (IgG anti -PF4/Heparin—quantification test) and aggregometry (a functional test—Multiplate^®^) should be performed [20].

Management in heparin-induced thrombocytopenia:

In cases of HIT suspicion or confirmation, heparin should be discontinued, and an alternative anticoagulant started (e.g., DTI) [20]. Patients in which HIT is confirmed should be anticoagulated for at least 4 weeks, extending for 3 months in associated thrombotic complications [28].

### 3.4. Ecmo Complications

#### 3.4.1. Thrombotic Events

Estimated incidence of thrombosis in adults under V-V ECMO range from 12.8% to 29% [1]. Circuit-related thrombosis is more frequent than patient-related thrombosis [1] and becomes clinically relevant when mechanical or functional membrane dysfunction requires circuit exchange or severe hemolysis emerges [11].

The goal of heparin therapy is reducing thrombin activity and fibrin formation, aiming to decrease thrombotic events without causing bleeding [8]. Some data confirm comparable thrombotic rates between standard and low anticoagulation protocols and showed a reduced incidence of bleeding events [1].

#### 3.4.2. Bleeding Events Management

**Figure 2:**
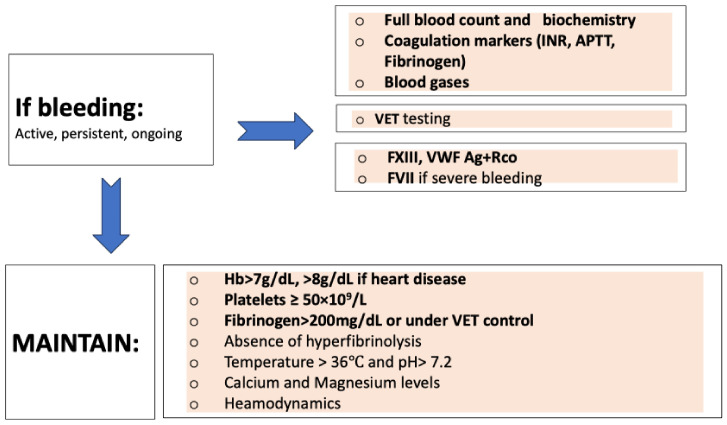
Coagulopathy management and transfusion support in the bleeding patient [29,30,31]. APTT: activated partial thromboplastin time; dL: deciliter; F: Factor; Hb: Hemoglobin; INR: international normalized ratio; mg: milligrams; VET: viscoelastic testing; VWF-Ag: von Willebrand factor Antigen; VWF-Rco: von Willebrand factor Ristocetin cofactor; >: more than; ≥ more or equal than.

If a bleeding event occurs, we recommend clinical evaluation and the following (Figure 2): full blood count, coagulation markers (including fibrinogen level), VET, FXIII, von Willebrand factor (VWF) (antigen-Ag and ristocetin cofactor-RCo), and FVII, according to bleeding severity. The rationale for assessing VWF (antigen-Ag and ristocetin cofactor-RCo) is to exclude acquired von Willebrand Syndrome (AVWS), measuring level and ratio.

It is recommended to measure FVII in case of severe bleeding since it is usually low in these cases; vitamin K or fresh frozen plasma can be administrated according to clinical status. Factor VIII measurement is not of value because it is usually high in inflammatory conditions, frequently seen in ECMO patients.

Uremia, creatinine, magnesium, and blood gases should be analyzed. At the same time, stabilization of clinical conditions is essential because of their important role in hemostasis control [29]. Thus, a temperature over 35–36 °C, pH over 7.2, ionized calcium over 1.2 mmol/L, normal magnesium level, and hemodynamic stabilization are recommended [30,31].

Hemoglobin (Hb) level should be within 7–9 g/dL and hematocrit >24–28% due to its role in primary hemostasis [29]. Cutoff of Hb <7.0 g/dL is considered for packed red blood cells [11].

Coagulopathy management should be based on VET analysis. Current guidelines and expert groups recommend the administration of platelet concentrate in cases of persistent bleeding associated with platelet count < 50 × 10^9^/L or <100 × 10^9^/L if brain injury and suggestion of platelet dysfunction by VET analysis [30,31].

In cases of low fibrinogen concentration (under VET analysis or <2 g/L in Clauss method) and active ongoing bleeding, fibrinogen concentrate should be considered at different doses, depending on the clinical setting and VET analysis [30,31]. Clauss’s method for fibrinogen level assessment can promote misinterpretation when hydroxyethyl starch is used to overcome blood loss or when DTI is used. In these cases, VET analysis is preferred.

A deficiency in FXIII leads to clot instability that is not hyperfibrinolysis-related [29]. FXIII deficiency can be suspected by VET analysis but needs confirmation of the exact level. In cases of ongoing or diffuse bleeding and low clot strength, despite adequate fibrinogen concentration, it is likely that a significant FXIII exists, and the administration of FXIII concentrate should be considered, or if FXIII is not available, replacement with fresh frozen plasma can also be performed [30,31].

These and other coagulation disturbances, along with hemorrhagic or thrombotic events, should be monitored and treated according to local algorithms for bleeding management, adapted to local circumstances and specific needs [30,31].

### 3.5. Monitoring Algorithm (Figure 3 and Figure 4)

○ Due to the possibility of using a bedside point of care (POC) test, with the advantage of fast test results and timely monitoring, we propose using either point of care aPTT or ACT for coagulation monitoring every 2 h or 4 h, according to patient status and difficulty to achieve the target (Figure 5).

**Figure 3:**
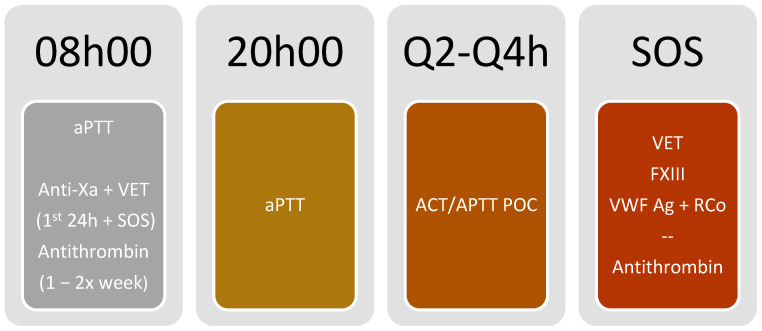
Monitoring protocol using unfractionated heparin anticoagulation. ACT: Activated clotting time; Anti-Xa: anti-activated factor X activity; aPTT: activated partial thromboplastin time; e: each; F: factor; POC: Point of care; VET: Viscoelastic testing; VWF:Ag: von Willebrand factor antigen; VWF:RCo: von Willebrand factor ristocetin cofactor; Our protocol is as follows: aPTT daily at each 08:00 and 20:00 h; antithrombin basal level and/or 1–2 times per week according to clinical situation; anti-Xa and VET only in the first 24 h of ECMO support and in SOS situations (ex: bleeding and thrombotic events or membrane changes); POC ACT or APTT daily in each Q2–Q4 h; VET, FXIII and VWF: Ag + RCo in SOS situations (ex: bleeding events), adding antithrombin level if thrombotic event or membrane change occurs.

**Figure 4:**
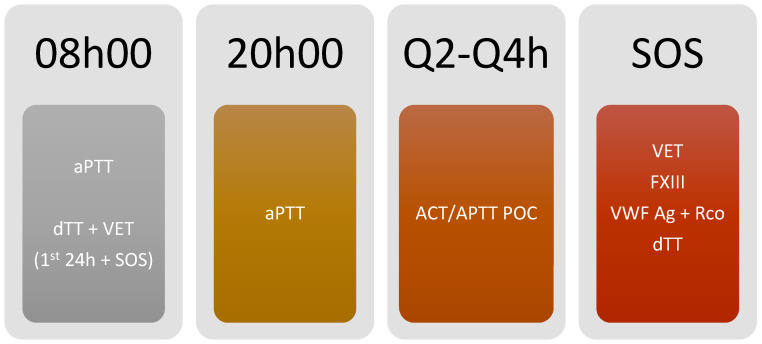
Monitoring protocol using bivalirudin anticoagulation. ACT: Activated clotting time; APTT: activated partial thromboplastin time; dTT: dilute thrombin time; F: Factor; POC: Point of care; VET: Viscoelastic testing; VWF:Ag: von Willebrand factor antigen; VWF:RCo: von Willebrand factor ristocetin cofactor; Our protocol is as follows: aPTT daily at each 08:00 and 20:00 h; dTT and VET only in the first 24 h of ECMO support and in SOS situations (ex: bleeding and thrombotic events or membrane changes); ACT daily in each Q2–Q4 h; VET, FXIII, VWF: Ag + RCo and dTT in SOS situations (ex: bleeding and thrombotic events or membrane changes).

**Figure 5:**
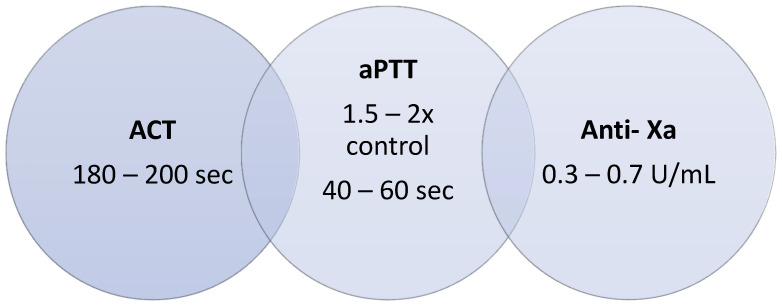
Anticoagulation targets for V-V-ECMO patients using multiple laboratory tests. ACT: Activated clotting time; aPTT: activated partial thromboplastin time; Anti-Xa: anti-activated factor X activity; sec: seconds; U: units; mL: milliliters.

How do we interpret and follow these recommendations when the results are discordant with each other? We suggest:

(A) Verify if these assays were performed 20 min after blood collection
(B) Verify if the blood was collected under the recommended conditions
(C) Verify if the ACT equipment is under adequate quality control and correct work conditions (reagents, instrument).
(D) These ranges can be reagent-dependent and must be checked by each laboratory
(E) Even if all previous items were ok, preferably follow the anti-Xa results and repeat all tests shortly after.

○ If POC methods are not available, aPTT should be monitored every 2 h or 4 h, according to patient status and difficulty in achieving the target.
○ If POC aPTT is not available, aPTT monitoring should be performed every 12 h, using a multimodal evaluation focusing on the anticoagulant effect.
○ Anti-Xa activity monitoring (using heparin) and dTT (for bivalirudin) should be performed in the first 24 h of monitoring and once daily.
○ In patients on heparin anticoagulation, AT levels should be measured regularly (once or twice a week according to clinical status—i.e., if suspecting heparin resistance or not able to achieve heparin effect), and supplementation with AT concentrate should be granted when deemed necessary.
○ When thrombotic complications occur, consider AT measurement. Maintain AT level over 80% (ideally between 80–120%).
○ Viscoelastic testing (ROTEM^®^, Quantra^®^) should be considered in the first 24 h of anticoagulation to have qualitative monitoring. Testing should be repeated when thrombotic or bleeding complications occur.
○ If there is a hemorrhagic event, FXIII, VWF (both antigen and functional), and FVII monitoring should be considered according to the volume of blood loss and the clinical scenario.
○ Heparin and bivalirudin dosages are adjusted according to a nomogram, as available in Table 3 and Table 4.

## 4. Discussion

During V-V ECMO support, patients’ blood is repeatedly exposed to an extracorporeal interface, implying permanent contact of blood with a non-biologic surface and promoting coagulation system activation that leads to a fragile balance between thrombosis and bleeding [1,31]. Overall, bleeding is more frequent than thrombosis [10,11]. Thrombosis and bleeding management during V-V ECMO should be tailored to the individual patient (personalized intervention) and account for the impact of its clinical condition (predictive intervention) [11]. Well-designed protocols in experienced ECMO centers should allow the safe eviction of anticoagulation in actively bleeding patients or patients with a very high risk for bleeding; additionally, management with low anticoagulation protocols in the case of high bleeding risk should be included in the protocol [1].

Undisputed and universally accepted anticoagulation practice in patients under ECMO support is still lacking, although periodically updated guidelines have been published, namely from ELSO. The inclusion of viscoelastic tests in the monitoring algorithm of the coagulation system in V-V ECMO will allow a more accurate characterization of the pathophysiology of bleeding or thrombosis. In our ECMO Referral Centre, we designed a coagulation management protocol that integrates recent laboratory testing methodologies [9,10]. With high-volume ECMO implementation, heparin-induced thrombocytopenia (HIT) and heparin resistance (HR) become major clinical issues. These were particularly evident in the context of severe SARS-CoV-2 infection [13]. In these patients, DTIs emerged as alternative agents, but doubts persist, mainly due to access limitations, lack of antidotes, high cost, and restrictive monitoring methods that hamper broad clinical implementation.

Each anticoagulation monitoring test has advantages and pitfalls [11]. It should be noted that no test allows the prediction of bleeding or thrombosis in patients under V-V ECMO support, often deriving contradictory data [12]. In a systematic review and meta-analysis that evaluated a strategy based on anti-Xa activity monitoring with other coagulation tests (ACT, aPTT, ROTEM, or TEG), some evidence emerged that suggested the occurrence of few bleeding events and eventually lower mortality rate, without increasing thrombotic events [12].

As shown in our recently published study, HemoCov, in severe COVID-19 patients, VET was very useful in identifying patients with worse prognosis, which showed more pronounced hypercoagulability and hypofibrinolysis [35]. VET tests achieve greater value for anticoagulation monitoring by establishing information on global hemostasis and are now increasingly used for bleeding management in different clinical scenarios [15,29]. We acknowledge our review has some caveats. First, we recognize that our conclusions are mostly based on studies and experiences of a single center. We acknowledge that well-designed, multicenter randomized trials evaluating complex coagulation monitoring strategies in patients under V-V ECMO are needed to define better goal-directed therapy and management. We tried to integrate the best and most recent evidence concerning anticoagulation management and monitoring in ECMO support. Second, we believe that the availability of viscoelastic tests and more complex monitoring algorithms is not universally established. This limits its recommendation, but cumulative evidence derived from studies and our center experience increases our understanding of the intricate relation between coagulation and extracorporeal circuits. Finally, we acknowledge that different populations might express different phenotypes that impact the coagulation system. To overcome this limitation, we cannot emphasize more strongly the need for additional studies.

## 5. Conclusions

Coagulation disorders remain one of the main problems that impact the outcome of patients under V-V ECMO. We propose a coagulation management protocol to tailor antithrombotic therapy prescription based on an algorithm that includes monitoring of anti-Xa, aPTT or ACT, AT levels, allied with more recent or emergent (non-conventional) viscoelastic tests [8,11]. This complex approach should translate into beneficial clinical intervention, with a more personalized and predictive approach to coagulation in patients undergoing V-V ECMO.

## Figures and Tables

**Table 1:** Causes of Heparin resistance and management [10,19].

Heparin Resistance Causes	Anti-Xa Activity	aPTT Level	AT Level	Management
**Pseudo-resistance**	Therapeutic	Low	Normal or Moderate/low	Adjust heparin dosage according to anti-Xa activity
**Low heparin concentration** (high clearance or increased binding to proteins)	Low	Low	Normal or Moderate/low	Increase UFH dosage or change to DTI
AT **deficiency**	Low	Low	Very low (<40–50%)	AT supplementation or change to DTI

Anti-Xa activity: Activity anti-activated factor X; aPTT: activated partial thromboplastin time; AT: Antithrombin; DTI: Direct Thrombin inhibitor.

**Table 2:** 4Ts score for diagnosis of Heparin-induced thrombocytopenia [25].

	Criteria	Points
Thrombocytopenia	Count drop > 50% Nadir ≥ 20 × 10^9^/L	2
	Count drop 30–50% Nadir 10–19 × 10^9^/L	1
	Count drop < 30% Nadir < 10 × 10^9^/L	0
Timing of the decrease in platelet count	5 to 10 days *	2
	>10 days or possible start in 5–10 days	1
	<4 days	0
Thrombosis manifestations	Acute thrombosis Cutaneous necrosis Systemic reaction	2
	Recurring thrombosis Other skin lesions Suspicion of thrombosis, no confirmation	1
	Asymptomatic	0
Other causes of thrombocytopenia	Apparently none (absence) Possible	2 1
	Confirmed	0

≤: lesser or equal than; >: more than; * If previous heparin exposure, consider: 2 points if thrombocytopenia ≤1 day, exposure less than 30 days ago; 1 points if thrombocytopenia ≤1 day, exposure 30 to 100 days.

**Table 3:** Heparin monitoring and adjustment using ACT ratio, APTT ratio, and anti-Xa [9,13,32,33].

ACT (s)	<140	141–160	161–179	180–200	201–240	241–270	>270
**UFH bolus (U/kg)**	20	10	-	-	-	-	-
**STOP UFH (min)**	-	-	-	-	-	30	60
**UFH adjustment**	+30%	+20%	+10%	-	−10%	−20%	−30%
**aPTT ratio**	**<1.2**	**1.21–1.30**	**1.31–1.49**	**1.5–2**	**2.01–2.25**	**2.26–2.49**	**>2.5**
**UFH bolus (U/kg)**	20	10	-	-	-	-	-
**STOP UFH (min)**	-	-	-	-	-	30	60
**UFH adjustment**	+20%	+15%	+10%	-	−10%	−20%	−30%
**Anti-Xa (units/mL)**	**<0.20**	**0.20–0.29**	**0.30–0.7**	**0.71–0.80**	**0.81–0.99**	**>1.00**	
**UFH bolus (U/kg)**	26	-	-	-	-	-	
**STOP UFH (min)**	-	-	-	-	-	60	
**UFH adjustment**	+4 U/kg/h	+2 U/kg/h	-	−1 U/kg/h	−2 U/kg/h	−3 U/kg/h	

ACT: Activated coagulation time; aPTT: activated partial thromboplastin time; anti-Xa: anti-activated factor X activity; Kg: Kilograms; min: minutes; s: seconds; U: unit; UFH: Unfractionated heparin.

**Table 4:** Bivalirudin monitoring and adjustment using ACT or APTT ratio [13,16,34].

ACT (s)	<180			180–200	200–250		>250
**Adjustment**	↑ 0.25–0.5 mg/Kg/h (if <140 s consider bolus 0.1–0.5 mg/Kg/h) → ↑ 10 to 20% each time			-	↓ dose 10 to 20%		↓ 30–50%
**STOP (min)**	-	-	-	-	-	-	30
**APTT ratio**	**<1.2**	**1.21–1.30**	**1.31–1.49**	**1.5–2.0**	**2.01–2.25**	**2.26–2.49**	**>2.5**
**Adjustment**	+20% (×1.2)	+10% (×1.1)		-	−10% (×0.9)		−50% (×0.5)
**STOP (min)**	-	-	-	-	-	30	30

ACT: Activated coagulation time; APTT: activated partial thromboplastin time; h: hour; Kg: Kilograms; mg: milligrams; min: minutes; s: seconds; ↓: decrease; ↑: increase.

## Data Availability

Not applicable.
